# Facial expressions of pain in cats: the development and validation of a Feline Grimace Scale

**DOI:** 10.1038/s41598-019-55693-8

**Published:** 2019-12-13

**Authors:** Marina C. Evangelista, Ryota Watanabe, Vivian S. Y. Leung, Beatriz P. Monteiro, Elizabeth O’Toole, Daniel S. J. Pang, Paulo V. Steagall

**Affiliations:** 1 Département de sciences cliniques, Faculté de médecine vétérinaire, Université de Montréal, 3200 rue Sicotte, Saint-Hyacinthe, Québec J2S2M2 Canada; 2 Groupe de recherche en pharmacologie animale du Québec, Université de Montréal, 3200 rue Sicotte, Saint-Hyacinthe, Québec J2S2M2 Canada; 3 Veterinary Clinical and Diagnostic Sciences, Faculty of Veterinary Medicine, University of Calgary, Calgary, Alberta Canada

**Keywords:** Health care, Translational research

## Abstract

Grimace scales have been used for pain assessment in different species. This study aimed to develop and validate the Feline Grimace Scale (FGS) to detect naturally-occurring acute pain. Thirty-five client-owned and twenty control cats were video-recorded undisturbed in their cages in a prospective, case-control study. Painful cats received analgesic treatment and videos were repeated one hour later. Five action units (AU) were identified: ear position, orbital tightening, muzzle tension, whiskers change and head position. Four observers independently scored (0–2 for each AU) 110 images of control and painful cats. The FGS scores were higher in painful than in control cats; a very strong correlation with another validated instrument for pain assessment in cats was observed (rho = 0.86, p < 0.001) as well as good overall inter-rater reliability [ICC = 0.89 (95% CI: 0.85–0.92)], excellent intra-rater reliability (ICC > 0.91), and excellent internal consistency (Cronbach’s alpha = 0.89). The FGS detected response to analgesic treatment (scores after analgesia were lower than before) and a cut-off score was determined (total pain score > 0.39 out of 1.0). The FGS is a valid and reliable tool for acute pain assessment in cats.

## Introduction

Pain management is frequently overlooked in cats and they are prescribed less analgesic drugs when compared with dogs^1–3^. This is due to challenges in feline pain recognition and assessment, lack of specific training in the subject and limited availability of pain assessment scoring tools in this species^3,4^.

Two validated behaviour-based pain assessment instruments have been published, the UNESP-Botucatu multidimensional composite pain scale^5^ and the Glasgow composite measure pain scale-feline (rCMPS-F)^6^. The latter has been updated and the definitive version included two features of facial expression (ears and muzzle), improving its discriminatory ability^7^. Although validated, each of these tools have their own limitations such as time-consuming implementation, validity tested for a single type of pain stimulus (i.e. ovariohysterectomy), and confounding effects of cats’ demeanour and drugs on pain scores^8–10^.

Along with the evaluation of behavioural changes, facial expressions have the potential to indicate emotional experiences in animals and provide valuable information regarding internal states^11^. Facial expressions can be a useful, valid and reliable tool for pain assessment in humans and other animals^12^. They can be objectively assessed using a facial action coding system (FACS) that measures the individual movements or ‘action units’ (AU) of the face that comprise an expression^13^. This system assigns independent codes to activity of individual muscles or groups of muscles. A feline-specific coding scheme (CatFACS) has been developed by studying the facial musculature of the domestic cat^14^.

Grimace Scales are simplified methods of assessing the facial expressions specifically related to pain. They were developed for mice^15^, rats^16^, rabbits^17^, horses^18^, sheep^19,20^, lambs^21^, piglets^22,23^ and ferrets^24^. Most of these scales consider four to five AU rated as absent, partially present or markedly present. Action units such as orbital tightening and ear position are listed across all species, however other facial features and some specific changes are different (i.e. flattening of the nose and cheek regions are observed in rats, in contrast to bulging in mice)^15,16^. For this reason, it is important to develop species-specific grimace scales^12^.

In cats, methods to quantify facial changes, focusing on linear distances between specific facial landmarks (i.e. distances between ears and muzzle) allowed distinction between painful and pain-free animals^25^. However, orbital tightening and whiskers position, that are commonly listed as action units in other species, were not included and a grimace scale for assessing pain in cats using facial expressions has not been published. More recently, a geometric morphometric approach has been described to study facial expressions of pain in cats. This approach has been proposed as basis for further application of machine learning algorithms for automated pain recognition^26^. On the other hand, grimace scales are simple and readily applicable in a clinical context, and the development of a new tool could improve feline pain management.

The widespread adoption of pain assessment scales requires testing of its validity (the ability of the scale to identify pain), responsiveness (ability to detect clinically important changes, such as worsening pain or an improvement following analgesic administration), and reliability (the overall reproducibility of the scores between and within raters)^27^. Additionally, to be useful as a clinical tool, an analgesic intervention threshold (cut-off score) should be determined to guide when analgesics administration is warranted^28,29^.

This study aimed to develop and validate the Feline Grimace Scale (FGS) to detect acute pain associated with naturally-occurring conditions (i.e. diseases causing somatic or visceral pain). More specifically, we aimed (1) to identify the facial features associated with pain in cats during development of the FGS; (2) to assess construct validity (including responsiveness), criterion validity and reliability of the FGS; and (3) to derive a cut-off score for rescue analgesia.

We hypothesized that (1) the FGS would be able to discriminate painful from non-painful cats in a clinical setting; (2) painful cats would score higher than non-painful cats (construct validity); the FGS scores would decrease after analgesic treatment (responsiveness); the FGS would correlate with the Glasgow rCMPS-F (criterion validity); the scores from different observers and from the same observer over time would be reliable (inter- and intra-rater reliability); and (3) a cut-off for rescue analgesia would be determined with high sensitivity and specificity.

## Methods

### Ethical statement

The study protocol was reviewed and approved by the institutional animal care and use committee of the Faculty of Veterinary Medicine, Université de Montréal (protocol number 17-Rech-1863). Experiments were conducted in accordance with the Canadian Council on Animal Care guidelines.

### Animals

Fifty cats of any age, breed and gender, admitted to the emergency and critical care unit of our veterinary teaching hospital (Centre hospitalier universitaire vétérinaire – CHUV) were recruited after owner’s written and informed consent. Another twenty healthy cats from the teaching colony of our institution were included as controls (non-painful cats).

Cats were excluded if they presented diseases or conditions that could affect facial expressions (e.g. ophthalmic conditions, head trauma, etc.), excessively shy and feral behaviour, or if they were administered sedatives and/or analgesics up to 24 hours before admission. Cats requiring immediate treatment (e.g. respiratory distress, severe hypovolemia, shock, active bleeding) were not recruited.

### Pain assessment and video-recording

**Figure 1:**
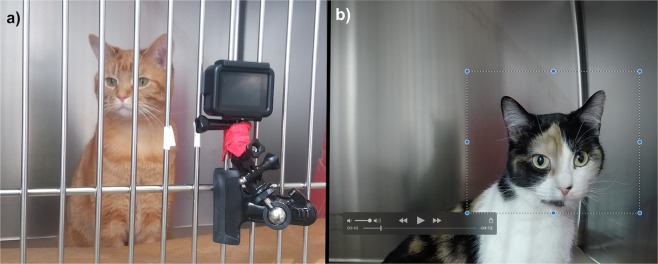
(**a**) Video-recording of cats. A wide-angle lens camera was placed between the cage bars and cats were video-recorded undisturbed for 6 min. **(b)** Image cropping and selection. Files were downloaded on to a computer and screenshots obtained from video-recordings.

Client-owned cats were recruited between March and November 2017. Upon presentation, full physical examination was performed, and cats were placed individually in cages for a five-minute acclimation period. Pain was assessed by one observer (MCE) according to the Glasgow composite measure pain scale for acute pain in cats (rCMPS-F)^6^. Briefly, this scale contains six questions regarding the overall cat’s appearance, response to interaction and palpation of a potentially painful area. Cats with scores of ≥4/16 were considered painful. Following the initial pain assessment, cats were filmed using a wide-angle high definition camera (GoPro Hero 5, GoPro, San Mateo, CA, USA) placed between the cage bars at the level of the eyes (Fig. 1a). Video-recording of each animal was performed for six minutes for later image selection and assessment. Hospital staff were not present, and cats were left undisturbed during video-recording. After video-recording, analgesics were administered to painful cats (treatment choice at the discretion of the responsible clinician) and the same procedure for pain assessment using rCMPS-F and video-recording was repeated one hour later.

Control cats were video-recorded between January and February 2017. The same video-recording procedure described above was performed with the control cats. A physical examination was performed, and pain was evaluated using the rCMPS-F. These cats were filmed twice, with a one-hour interval (no analgesic treatment was given).

### Image capture selection and development of the FGS

Image selection and assessment of facial expressions were performed by one observer (MCE) using screenshots obtained from video-recordings (QuickTime Player, version 10.4, Apple Inc., Cupertino, CA, USA) when the cat was facing the camera, but not sleeping, grooming or vocalizing (Fig. 1b). From each six-minute video, one image was selected from every two-minute interval and the best of three images (based on image quality) was chosen. The images were cropped to include only the cats’ face and part of the shoulders. This observer was not blinded to the time point of the video (before or after analgesia or one-hour interval) during the image selection; however, the observer did not have access to their corresponding rCMPS-F scores.

**Figure 2:**
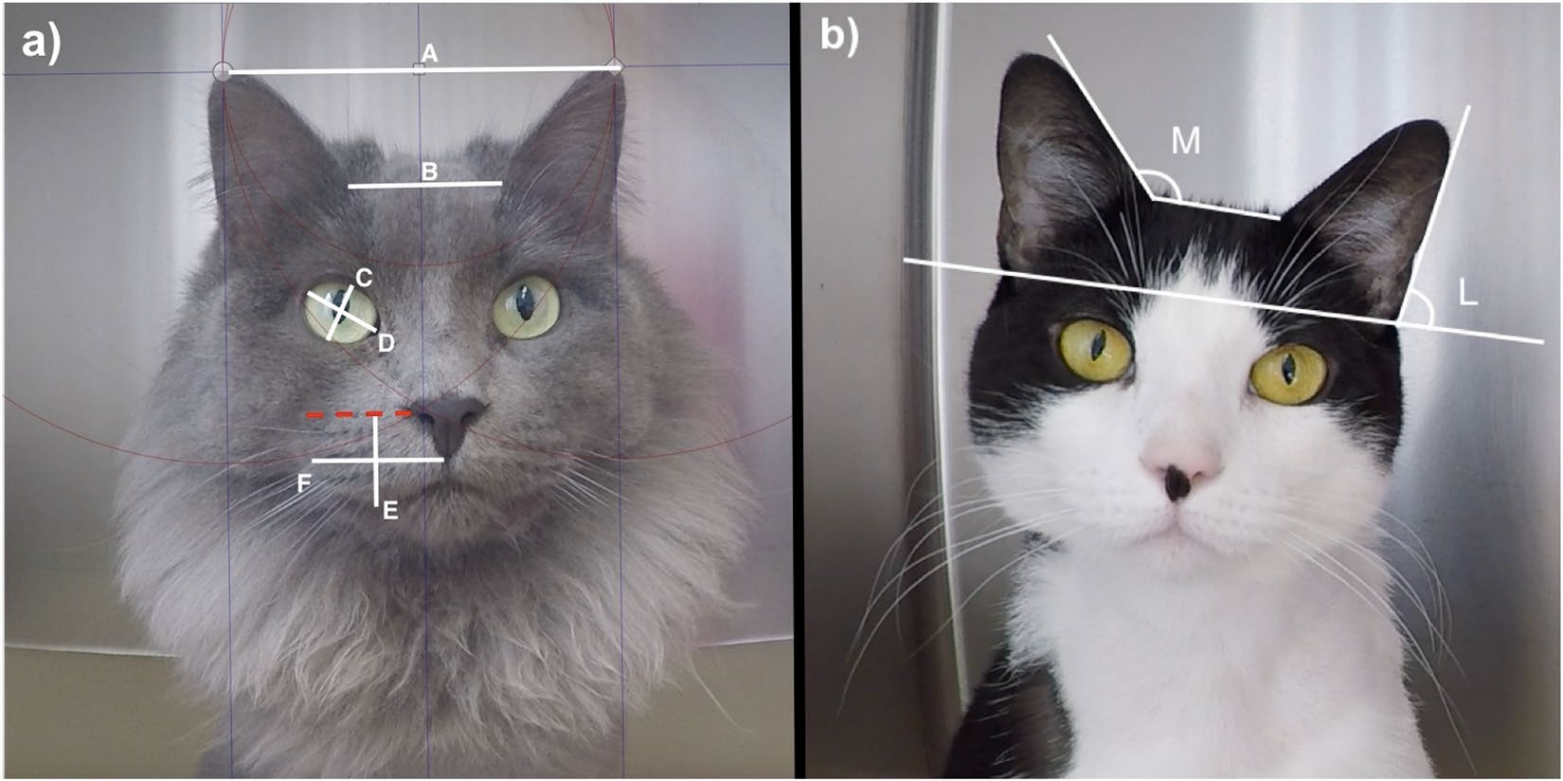
**(a)** Measured distances for the ears, eyes and muzzle. A - Ears: tip to tip; B - Ears: base to base; C - Eye: height; D - Eye: width; E - Muzzle: height; F - Muzzle: width. Eyes and muzzle distances were measured bilaterally and averaged. **(b)** Measured ear angles. M - Medial angle: between the medial border of the ear and top of the head; L - Lateral angle: between the lateral border of the ear and an imaginary line connecting both marginal cutaneous pouches. Angles were measured bilaterally and averaged.

Two individuals (MCE and RW) visually compared images (thumbnails and full-size pictures) of 20 controls and 31 client-owned painful cats (rCMPS-F ≥ 4/16) before the administration of analgesics to identify any differences in facial features between these two groups. Features that were consistently different between both groups were listed as action units (AU) and used to create the FGS. The AU were illustrated and described in a manual that was later used for training purposes (Appendix 1; Supplementary Material). In order to corroborate these findings, distances between pairs of landmarks were measured (PixelStick, version 2.10.1, PlumAmazing Softwares, Princeville, HI, USA) and the ratios between two pairs were calculated (distances between the ear tips and ear bases, eye height and width, muzzle height and width; adapted from^25,30^) (Fig. 2a). Ear angles were also measured (Screen rulers for Mac, version 1.13.1, Ondesoft Softwares, Venice, CA, USA) (Fig. 2b). These measurements were performed on 51 images (20 controls and 31 client-owned painful cats before analgesia) by the main observer (MCE), not blinded to the groups. A second observer (RW), blinded to the groups, independently repeated the measurements in a randomly chosen sample of one third of the images using a random number generator. The agreement between both observers was calculated.

### Image scoring and validation of the FGS

Four observers, blinded to the groups and time when the images were obtained (VSYL, BPM, PVS and DSJP; two PhD candidates and two board-certified veterinary anaesthesiologists) were supplied with the training manual. They independently scored 110 images from 55 cats (20 controls and 35 client-owned, before and after analgesic treatment or one-hour interval), twice 30 days apart. The images were numerated using a random sequence generator (www.randomization.com) by one investigator (MCE) and a different sequence was generated for each round. An online questionnaire (SurveyMonkey, San Mateo, CA, USA) was built with one image per page and five questions regarding each of the AU: ears, eyes, muzzle, whiskers and head position. For each AU, the observers could select one of the four following options: 0 = AU is absent; 1 = moderate appearance of the AU, or uncertainty over its presence or absence; 2 = obvious appearance of the AU; or “not possible to score” (e.g. if the AU was not clearly visible). Image scoring was performed between February and April 2018.

A single total pain score per image was calculated as the sum of scores from each AU divided by the maximum possible score (e.g. 4/10 = 0.4), excluding the AU marked as “not possible to score”. The final FGS score ranged from 0 to 1. Images receiving more than two “not possible to score” from the same rater were excluded from the analysis.

### Statistical analysis

Statistical analyses were performed using the SPSS software (version 24.0.0.1, IBM SPSS Statistics, Armonk, NY, USA).

#### Development of the FGS

Bonferroni-corrected independent t-tests were used to compare linear measurements (mean distance ratios and angles) between controls and painful client-owned cats.

Intraclass correlation coefficients (ICC) were calculated based on single measures (ICC_single_) and average of measures (ICC_average_) when indicated, using two-way random effects model for absolute agreement between the linear measurements (distance ratios and angles) performed by the two observers.

#### Validation of the FGS

The effect of sex on FGS scores was assessed using a linear mixed model with group as between-subject factor and time as within-subject factor, using sex as a cofactor.

Construct validity (by known-groups discrimination) was assessed based on hypothesis testing. The hypothesis was that painful cats would score higher than non-painful ones and Mann-Whitney U test was used to compare the scores from both groups. Responsiveness, the sensitivity to change (as part of construct validity), was assessed based on the hypothesis that FGS scores for painful client-owned cats before analgesia would be higher than those assessed after analgesic treatment and the scores would not change in control group after the 1 h interval. Wilcoxon signed rank tests were used to compare the scores within each group. The average of the scores given by four observers unaware of the groups and time points was used. A p value < 0.05 was considered significant.

Criterion validity, the correlation with a gold standard (rCMPS-F), was assessed using Spearman’s rank correlation between FGS and rCMPS-F scores provided by the main observer (MCE) and interpreted as following: rho < 0.19 = very weak; 0.20–0.39 = weak; 0.4–0.59 = moderate; 0.6–0.79 = strong; 0.8–1.0 = very strong^31^.

Reliability between raters (inter-rater) and by a single rater over time (intra-rater) were assessed with an ICC, calculated for each of the four observers independently assessing the same image and a single rater (comparing scores assigned to the same images on two rounds, 30 days apart). For inter-rater reliability, a two-way random effects ICC model for absolute agreement was used (calculated for both rounds 1 and 2). For intra-rater reliability, a two-way mixed effects ICC model, for absolute agreement was chosen. Interpretation was based on the ICC _single_ as following: ICC < 0.5 = poor, 0.5–0.75 = moderate, 0.75–0.9 = good, and >0.90 = excellent reliability^32^.

The agreement between the scores from the main observer (MCE) and the average of the four raters was calculated using the Bland and Altman method^33^.

Internal consistency was assessed with Cronbach’s alpha coefficient calculated for the final FGS score and for each AU based on the scores of the main observer (MCE), recalculating the coefficient with each AU deleted. Interpretation was performed as following: alpha < 0.65 = unsatisfactory; 0.65–0.69 = fair; 0.7–0.74 = moderate; 0.75–0.79 = good; >0.8 = excellent^34^.

The analgesic threshold (cut-off for rescue analgesia) was determined with a receiver operating characteristics (ROC) curve analysis. The ability of the FGS to discriminate between absence (rCMPS-F < 4 = no pain) and presence of pain (rCMPS-F ≥ 4 = pain) was assessed by comparing the area under the curve (AUC) generated from the scores of the main observer (MCE) with an AUC of 0.5. An AUC between 0.50–0.70 represented low accuracy; between 0.70–0.90 = moderate accuracy; and for AUCs over 0.90 = high accuracy^35^.

## Results

### Development of the FGS

**Figure 3:**
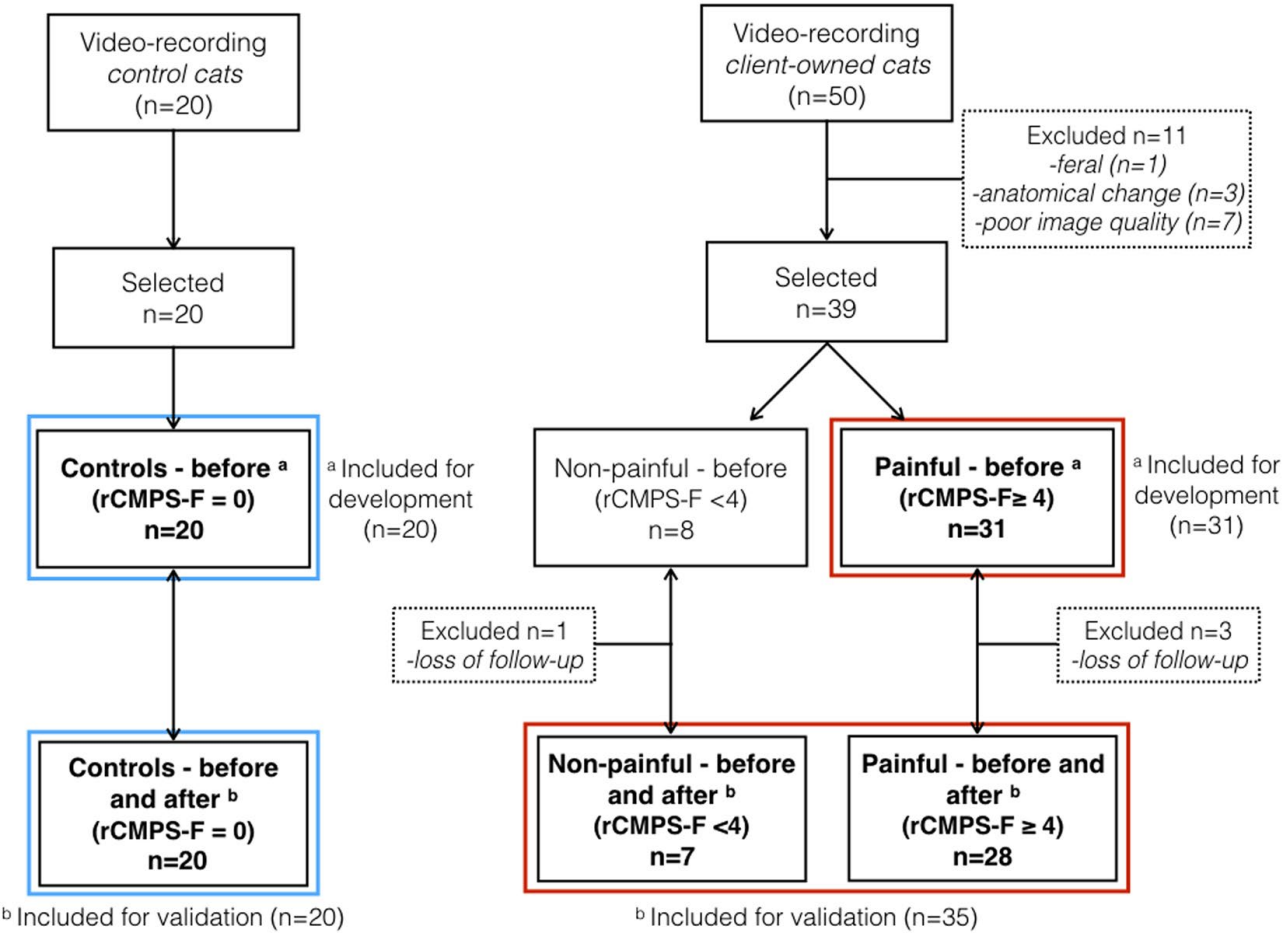
Flowchart of the animals included in the study. Blue boxes = control cats included for: ^a^development and ^b^validation of the Feline Grimace Scale (FGS). Red boxes = client-owned cats included for: ^a^development and ^b^validation of the FGS.

Client-owned cats: 50 adult cats of various breeds (24 females, 26 males) were enrolled in the study. Most cats presented with abdominal pain (diagnoses included hepatic lipidosis, cholangitis, pancreatitis, inflammatory bowel disease, suspected foreign body, lymphoma, constipation, urethral obstruction, urolithiasis and idiopathic cystitis). Eleven cats were excluded (Fig. 3). Landmarks for measurements could not be identified in black cats (n = 2, comprised within poor image quality). Finally, 31 cats [domestic short haired (n = 17), domestic long-haired (n = 7), Siamese (n = 2), Cornish Rex (n = 1), Bengal (n = 1), Maine Coon (n = 1), Savannah (n = 2)] presented rCMPS-F scores ≥ 4/16 [median (range): 5 (4–8)] and their images were included in the development of the FGS (14 females, 17 males; mean ± SD age 6.3 ± 3.6 years and weight 5.6 ± 1.9 kg) (Fig. 3).

Control cats: All of the 20 cats [domestic short-haired (n = 17), domestic long-haired (n = 3)] presented rCMPS-F scores = 0 [median (range): 0 (0–0)] and were included in image assessment (15 spayed females, 5 castrated males; mean ± SD age 3.1 ± 1.1 years and weight 3.8 ± 0.5 kg) (Fig. 3).

Both evaluators agreed on their visual assessment. Action units were defined as follows: (1) Ear position: refers to the tips of ears pulled apart and rotated outwards; (2) Orbital tightening: narrowing of the orbital area, with a height between eyelids smaller than 50% of eyes width, or tightly closed eyelid (squinted eyes); (3) Muzzle tension: flattening and stretching of the muzzle from round to an elliptical shape (muzzle may be bulged); (4) Whiskers position: movement of whiskers forward (rostrally and away from the face), as if standing on end (spiked); (5) Head position (in relation to the shoulders): head below the shoulder line or tilted down (chin toward the chest) (Appendix 1; Supplementary Material).

Linear distance ratios and angles were significantly different between groups (Table 1). The agreement between the measurements of the two observers (MCE and RW) was good for ears ratio and medial ear angle, and excellent for the eyes ratio, muzzle ratio and lateral ear angle. Ears ratio - ICC_single_ = 0.76 (95% CI: 0.46–0.90); Eyes ratio - ICC_single_ = 0.97 (95% CI: 0.92–0.99); Muzzle ratio - ICC_single_ = 0.91 (95% CI: 0.78–0.97); Medial angle - ICC_single_ = 0.77 (95% CI: 0.48–0.91); Lateral angle - ICC_single_ = 0.91 (95% CI: 0.76–0.96).

**Table 1:** Calculated ratios for the ears, eyes and muzzle, and measured ear angles to discriminate control and client-owned painful cats.

Measurements	Control (n = 20)	Painful (n = 31)	p value
Ear tips/bases ratio	2.85 ± 0.3	2.34 ± 0.3	p < 0.001
Eyes height/width ratio	0.79 ± 0.1	0.50 ± 0.2	p < 0.001
Muzzle height/width ratio	0.70 ± 0.1	0.50 ± 0.1	p < 0.001
Medial ear angles	126.5 ± 4.7°	140.4 ± 6.5°	p < 0.001
Lateral ear angles	78.9 ± 3.1°	68.5 ± 5.9°	p < 0.001

Data are presented as mean ± SD. Ratios were calculated between the distances of ear tips/bases, eyes height/width and muzzle height/width. Angles are presented in degrees. Excluding the distances between ear tips and bases, other measurements were performed bilaterally and averaged. Means across groups were compared using Bonferroni-corrected independent t-tests.

### Validation of the FGS

Images from 35 client-owned [domestic short haired (n = 21), domestic long-haired (n = 8), Siamese (n = 2), Cornish Rex (n = 1), Bengal (n = 1), Maine Coon (n = 1), Savannah (n = 1)] and 20 control cats (before and after analgesic treatment or one-hour interval) were included for the validation of the FGS. Seven out of the 35 client-owned cats presented rCMPS-F scores < 4/16 and 28 presented scores ≥ 4/16 (16 females, 19 males; mean ± SD age 6.8 ± 3.8 years and weight 5.8 ± 2.2 kg) (Fig. 3). Sex did not produce a significant effect on FGS scores (linear mixed model; p = 0.63).

Median (range) time to complete the survey was 78 (33–103) minutes and 63 (32–71) minutes for rounds 1 and 2, respectively. The AU whiskers position had the highest percentage of “not possible to score” answer, representing 10.2% of the images whereas the incidence of “not possible to score” selections for muzzle, head, ears and eyes was 3.6%, 2.7%, 0.22% and 0%, respectively.

#### Construct validity

**Figure 4:**
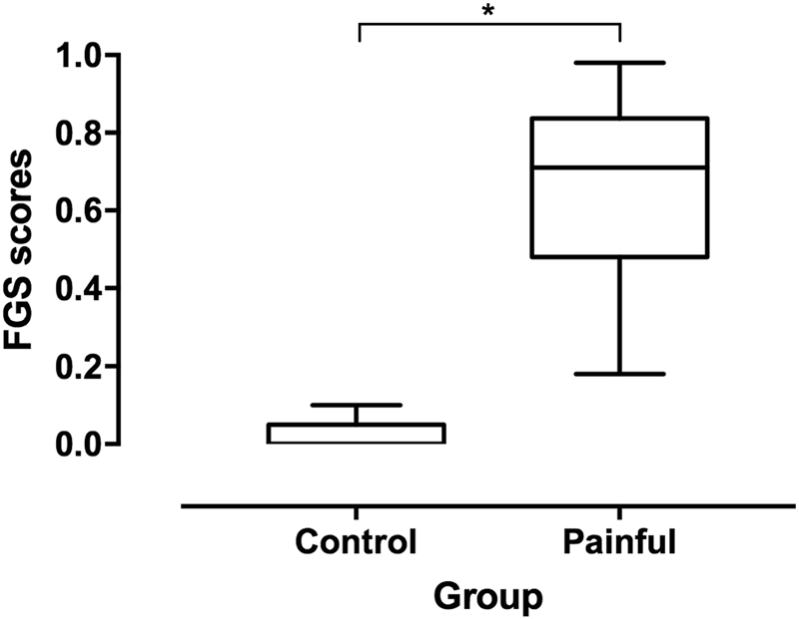
Boxplot showing median (solid line) and interquartile ranges for Feline Grimace Scale (FGS) scores in control (n = 20) and painful cats (n = 28). The whiskers represent the range. The FGS scores were higher for painful than control cats (Mann-Whitney U test, *p < 0.001).

Known-groups discrimination was used to assess construct validity. The FGS scores were higher in client-owned painful (before analgesia) than in control cats [median (range): 0.71 (0.18–0.98) and 0 (0–0.1), respectively (Mann-Whitney U test, p < 0.001; Fig. 4)].

#### Responsiveness

**Figure 5:**
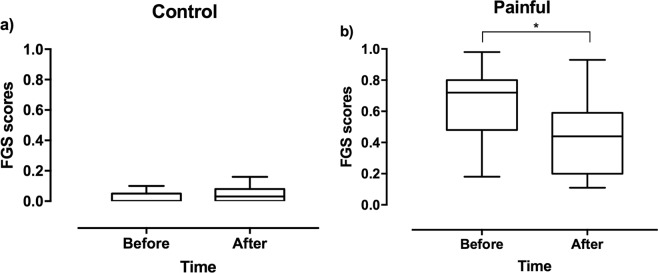
(**a)** Boxplot showing median (solid line) and interquartile ranges for Feline Grimace Scale (FGS) scores in control cats (n = 20). The FGS scores did not change for control cats after 1 h (without treatment; Wilcoxon signed rank test, p = 0.342). (**b**) Boxplot showing median (solid line) and interquartile ranges for FGS scores in painful cats (n = 15). The FGS scores decreased in painful cats after analgesia (Wilcoxon signed rank test, *p = 0.003).

For the responsiveness assessment, data from 15 painful cats before and after analgesia were available. Different analgesic drugs, doses and routes of administration were used: butorphanol (n = 9), buprenorphine (n = 4), hydromorphone (n = 1), meloxicam (n = 1). The FGS scores did not change in the control group after the one-hour interval (without any treatment) [median (range): 0 (0–0.1) and 0 (0–0.16); Wilcoxon signed rank test, p = 0.342], but a significant decrease was observed in painful cats after analgesic treatment [0.72 (0.18–0.98) and 0.44 (0.11–0.93); Wilcoxon signed rank test, p = 0.003] (Fig. 5). Similarly, rCMPS-F scores did not change in the control group [median (range) before: 0 (0–0) and after: 0 (0–0), Wilcoxon signed rank test, p = 1.0] and significantly decreased in painful cats after treatment [before: 5 (4–8) and after 3 (0–10), Wilcoxon signed rank test, p = 0.006].

#### Criterion validity

**Figure 6:**
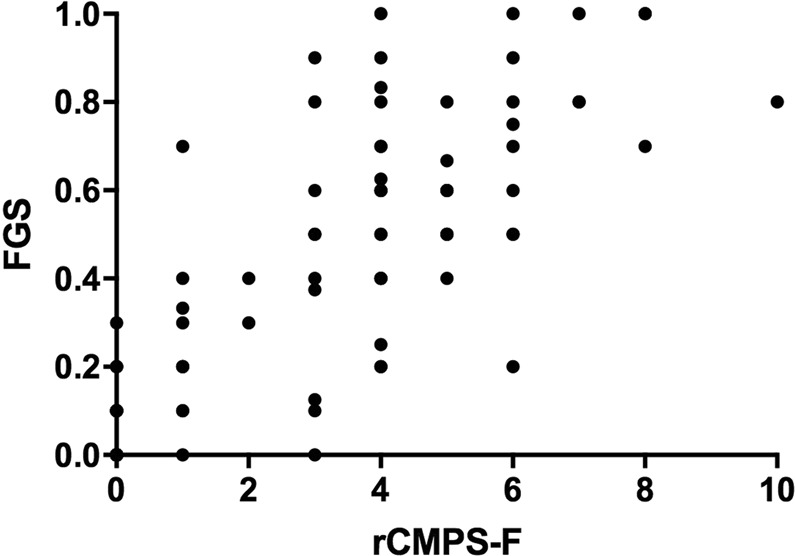
Scatterplot showing correlation between Feline Grimace Scale (FGS) and Glasgow composite measure pain scale for acute pain in cats (rCMPS-F) scores (Spearman’s rho = 0.86, p < 0.001) from control (n = 20) and client-owned cats (n = 35).

A very strong correlation was observed between the rCMPS-F and FGS (rho = 0.86; p < 0.001). A total of 110 images from both groups (control, n = 20 and client-owned, n = 35) and both time points (before and after) were considered for this analysis (Fig. 6).

#### Reliability

Inter-rater reliability for FGS scoring was good during both round 1 and 2 [ICC_single_ = 0.89 (95% CI: 0.85–0.92) and ICC_single_ = 0.89 (95% CI: 0.84–0.92), respectively]. Inter-rater reliability of individual AU ranged from moderate (muzzle and whiskers) to good (ears, eyes and head position) (Table 2).

**Table 2:** Inter-rater reliability of the Feline Grimace Scale.

Action unit		Round 1 ICC (95% CI)	Round 2 ICC (95% CI)
Ears	ICC_single_	0.87 (0.82–0.90)	0.85 (0.81–0.89)
	ICC_average_	0.96 (0.95–0.97)	0.96 (0.94–0.97)
Eyes	ICC_single_	0.86 (0.81–0.89)	0.83 (0.77–0.88)
	ICC_average_	0.96 (0.95–0.97)	0.95 (0.93–0.97)
Muzzle	ICC_single_	0.63 (0.53–0.72)	0.67 (0.58–0.75)
	ICC_average_	0.87 (0.82–0.91)	0.89 (0.85–0.92)
Whiskers	ICC_single_	0.55 (0.43–0.66)	0.60 (0.48–0.70)
	ICC_average_	0.83 (0.75–0.89)	0.86 (0.79–0.90)
Head position	ICC_single_	0.90 (0.87–0.93)	0.88 (0.84–0.91)
	ICC_average_	0.97 (0.96–0.98)	0.97 (0.96–0.98)
Final FGS score	ICC_single_	0.89 (0.85–0.92)	0.89 (0.84–0.92)
	ICC_average_	0.97 (0.96–0.98)	0.97 (0.96–0.98)

Intraclass correlation coefficient (ICC) estimates and their 95% confidence intervals (95% CI) were calculated based on single measures (ICC_single_) and average (ICC_average_) of measures (raters = 4), using two-way random effects model for absolute agreement. Interpretation was performed based on ICC_single_ as following: ICC < 0.5 = poor reliability, between 0.5 and 0.75 = moderate reliability, between 0.75 and 0.9 = good reliability, and >0.90 = excellent reliability^32^.

Intra-rater reliability was excellent for all observers 30 days after the first round of scoring. Observer 1: ICC_single_ = 0.91 (95% CI: 0.81–0.95); observer 2: ICC_single_ = 0.94 (95% CI: 0.91–0.96); observer 3: ICC_single_ = 0.95 (95% CI: 0.93–0.97); observer 4: ICC_single_ = 0.92 (95% CI: 0.88–0.94).

The scores from the main observer (MCE) and the average of the four raters had strong agreement, minimal bias (0.0047) and narrow limits of agreement (−0.18 to –0.19) (Appendix 2; Supplementary Material Fig. S1).

#### Internal consistency

Cronbach’s alpha coefficient calculated for the final FGS score was 0.89, indicating excellent internal consistency. The recalculated coefficients by deleting each AU were: ears (alpha if deleted = 0.86), eyes (alpha if deleted = 0.90), muzzle (alpha if deleted = 0.85), whiskers (alpha if deleted = 0.88), head position (alpha if deleted = 0.86). The alpha was minimally affected by removing any single AU, thus all AU contributed similarly to the final score.

#### Analgesic threshold

**Figure 7:**
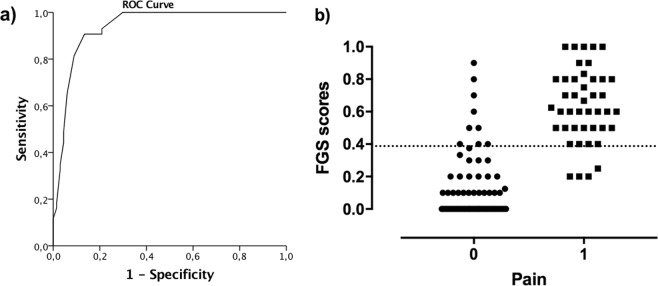
**(a)** Receiver operating characteristics (ROC) curve showing the cut-off point >0.39 for rescue analgesia, with sensitivity of 90.7%, and specificity of 86.6%. The area under the curve (AUC) of 0.94 (95% CI: 0.89–0.98), represents high accuracy of the Feline Grimace Scale (FGS). **(b)** Scatterplots showing the FGS scores and cut-off point for rescue analgesia (0.39), identified from the ROC curve analysis. N = 110 images of cats’ faces were classified as “absence of pain (0)” or “presence of pain (1)” according to Glasgow composite measure pain scale for acute pain in cats (rCMPS-F) scores.

The classification of 110 images according to the rCMPS-F scores resulted in 43 considered as “presence of pain” and 67 as “absence of pain”. The ROC curve was originated by plotting the true positive rate (sensitivity) against false positive rate (1 – specificity). The AUC of 0.94 (95% CI: 0.89–0.98) with p < 0.001 indicated a high discriminative ability (high accuracy) for the FGS (Fig. 7). The cut-off score of 0.39 (from a maximum of 1.0) was selected for representing an optimal balance between sensitivity (90.7%) and specificity (86.6%) (Appendix 2; Supplementary Material Table S1).

## Discussion

This study reported the development and validation of the FGS in the clinical setting using image assessment. A diverse sample of cats was included, with cats presenting pain from different sources and intensities. The FGS was developed by comparing the facial features of control and painful cats, then its validity and reliability were tested using images obtained from the video-recordings.

Two distinct populations of cats (healthy control cats and client-owned painful cats) of various breeds were studied. There were more females than males in the control group, but both genders were equally represented within the client-owned group, besides, there was no effect of sex on FGS scores. Sex and strain differences in laboratory animals have been previously reported in pain research, hence the importance of including both sexes in the development of a pain scale^36^. Although different breeds were represented within our client-owned population, most of the cats were domestic short-haired. Brachycephalic breeds were not included. Indeed, one Persian and one Himalayan were initially recruited, however they were excluded from final analysis due to poor image quality. Morphological differences (round-shaped skulls and decrease in facial width) related to breed-specific features have been previously observed in brachycephalic cats^37^. At this point it is not known if brachycephalic cats present the same AU related to pain as mesocephalic and dolichocephalic cats, and if these changes could present a source of bias in the FGS.

Five AU (ear position, orbital tightening, muzzle tension, whiskers position and head position) were identified. Similar AU have been previously described in mice^15^, rats^16^, rabbits^17^, horses^18^ and ferrets^24^, and head position has been described in sheep^20^.

These AU described on the FGS are consistent with ear action descriptors: “ears flattener”, “ears downwards”, and action units: “eye closure”, “nose wrinkler and upper lip raiser”, “whiskers retractor” and “whiskers protractor” presented on CatFACS^14^. They are also consistent with a geometric morphometric study that identified changes in the feline facial shape after a painful stimulus including “a more lateral and ventral positioning of the ears”, “a slightly narrowed eye aperture” and “reduced distance between the cheeks, mouth and nose region”^26^.

The visual comparison of the images for the development of the FGS was performed based on previous work on the development and validation of the Mouse, Rat, Rabbit, Horse, Sheep and Lamb Grimace Scales^15–20^ where similar methodology of comparison of two distinct group of animals were described. The demonstration of construct validity in the results supports the methods used in this scale development, though we acknowledge that alternative, less subjective methods (such as the study of the anatomy of facial musculature structure in cadavers^24^ or species-specific facial coding schemes^14^) are now available. In addition to the visual comparison of the images, distances and angles were measured as an additional quantitative outcome to corroborate our findings. These measurements were used in the description of the AU for the training manual. A second observer independently repeated the measurements in a randomly selected sample of one third of the images to avoid bias in measurements, and the agreement between their measures was good to excellent. The ratios between two distances rather than the actual measures were considered for group comparisons, to account for differences in the distance between the cat and the camera. According to our results, the eyes’ height was approximately 80% of its width in control cats and approximately 50% in the painful ones (when eyes are partially closed or squinted). Similar results were observed in lambs undergoing tail docking^21^. Likewise, the muzzle height decreased from nearly 70% of its width to 50% in the presence of pain. The medial ear angle increases and the lateral decreases as the ears flattens in painful cats. Distances between two pairs of landmarks in the cats’ ears and muzzle were previously reported as significantly different between painful and pain-free cats^25^.

The assessment of facial expressions in laboratory animals has been performed using still images (screenshots obtained from video recordings)^15,16^. We used similar methodology, where the cats were video-recorded undisturbed in their cages. Animals were free-ranging and able to express their natural behaviours. Other studies included photographs taken when the animals were restrained^19,22,24,25^. However, physical restraint significantly affected facial expressions scores in lambs^21^. Avoiding handling or close contact with the animal during image acquisition has been suggested, and the authors argue that leaving an animal to perform the behaviours in conditions that meet their needs is likely to yield the best results during the development of facial expression scales^12^.

In our study, black cats were excluded due to lacking image quality and difficulty in identifying landmarks in their faces. Similar difficulties were reported when scoring dark coated horses’ faces^18^. These issues may be resolved using high definition cameras or real-time scoring, which has been demonstrated to be possible in rats^38^. In a recent study, our research group tested real-time scoring using the FGS and reported good agreement (small bias and narrow limits of agreement) with image assessment^39^.

The FGS showed high discriminative ability between painful and non-painful cats. Construct validity assesses whether the tool is measuring something (a construct) that cannot be directly observed (e.g. pain)^27^. Known-groups discrimination was the method chosen to confirm the construct validity through hypothesis testing. This approach is in agreement with the validation of the Mouse and Rat Grimace Scales^15,16^ and behaviour-based feline pain scales^5,6^. For responsiveness assessment, different analgesic drugs, doses and routes of administration were used. Even with such a diverse sample receiving different analgesic drugs and dosage regimens, the FGS detected the response to analgesic treatment in painful cats. Correspondingly, the scores in the control group did not change after one hour, however, these cats did not receive any sham analgesia or handling. This represents a limitation and the impact of the physical experience of drug administration was not accounted. In a follow-up study, it would be important to determine the effect of specific analgesic drugs using fixed dosage regimens (including sham analgesia) after a standardised painful stimulus to confirm these findings.

Criterion validity was tested using concurrent validation of a new scale and a ‘gold standard’^27,40^. In the absence of a gold standard when evaluating pain, a validated pain scale for cats (rCMPS-F)^6^ was used. Similar approaches have been applied in laboratory rodents, where the criterion standard was mechanical hypersensitivity testing^15,41^. The most recent version of the rCMPS-F^7^ was not used since it includes two features of facial expressions that could bias our results. Concurrent validation was shown by correlating the FGS with rCMPS-F scores and a very strong correlation was observed. Additionally, the rCMPS-F scores were used to determine the presence of pain (rCMPS-F ≥ 4) or its absence (rCMPS-F < 4) for the ROC curve analysis. The drawback of this approach is how to be sure that the animals were in pain and not stressed. To reduce the bias, excessively shy and feral cats were excluded, knowing that demeanour influences the scores of feline-specific pain scales^10^. Future studies should investigate how changes in demeanour impacts the FGS.

The FGS showed good overall inter-rater reliability (ICC_single_ = 0.89) and excellent intra-rater reliability (ICC_single_ > 0.91). Our results are similar to those reported for mice, ICC = 0.90^15^; rats, ICC = 0.90^16^/ ICC_single_ = 0.85^29^ and horses, ICC = 0.92^18^. Interpretation was performed based on the ICC estimate for single measures (ICC _single_). The choice of reporting the estimates based on a single measure or the average of k measures depends on how the scale will be applied in a clinical context (e.g. if a decision will be made based on the scores of a single observer or on the average of a number of observers). The ICC_average_ is usually higher and ideally, both estimated should be reported along with their confidence interval 95%, as reported herein^32^.

A neglected area in grimace scale research is the role of rater training. It is currently unclear to what extent training is important as most papers do not describe if training has taken place^42^. The raters in our study have years of experience working with cats. It is unknown, and deserves further investigation, how reliability would be affected by novice raters.

The internal consistency of the FGS was excellent. This result agrees with those reported for the Mouse and Rat Grimace Scales (alpha = 0.89^6^ and 0.84^29^, respectively). The Cronbach’s alpha must be interpreted with caution, as the value will be higher for longer scales^27^. However, interpretation was performed according to the guidelines for scales with 6 items or less and a sample size between 100 and 300^34^.

The clinical utility of a pain scale is improved when an objective cut-off (or interventional) score informs the need for analgesia. For the FGS, the score for rescue analgesia is >0.39 out of 1.0. The cut-off score is a guide to help with treatment decisions and other values can be adopted depending on desired sensitivity and specificity. Similar methodology based on the ROC curve analysis was used for the validation of the UNESP-Botucatu multidimensional composite pain scale for cats^5^, Rat Grimace Scale^29^ and a Sheep Pain Facial Expression Scale^19^. Further studies are warranted to corroborate this finding in the clinical setting, using real-time assessment.

This study has some limitations: (1) It was an observational study, the decision for analgesic treatment was made by the clinician in charge. The observer did not interfere with the clinical judgement. It would be pertinent to test the FGS’s performance in a controlled interventional study, using the cut-off score for analgesic treatment determined by the ROC curve. (2) Image selection from the video recordings and the development of the scale were performed by the same observer (MCE). This observer was not blinded to the groups or time points of the video (before or after) during the image selection. Additionally, image selection took place three months before the beginning of the scoring sessions. Our research group is carrying out another study addressing this limitation, where image selection is performed by an independent observer. (3) No power analysis or sample size calculation was performed before the beginning of the study since it is not possible to estimate the percentage of client-owned cats that would be presented with pain. There is no consensus to define sample size in studies involving the development of pain scales with the same rigor as found in controlled trials^43^. Some grimace scales were developed using a within-subject design. In this setting, fewer subjects are required and the animal is evaluated before and after the induction of a standardised painful stimulus, which was the case in mice, rats, horses, sheep and ferrets^15,16,18,20,24^. In contrast, other studies used a between-subject design (case-control studies) to identify dissimilar facial features and behaviours associated with naturally-occurring painful diseases in cats and sheep^6,19,25^. (4) The lack of a baseline from the same animal is a limitation, and within-, rather than between-subject design is preferable^12^, however, to account for the variability among individuals we included a larger number of animals, similar to the population size used in the development of a Sheep Pain Facial Expression Scale^19^.

In conclusion, the FGS demonstrated high discriminative ability, a very high correlation with another validated instrument for pain assessment in cats, good overall inter-rater reliability, excellent intra-rater reliability, and excellent internal consistency. Furthermore, the FGS detected the response to analgesic treatment and a cut-off score was determined, making this a potential practical tool in both research and clinical settings. The FGS is a valid and reliable tool for acute pain assessment in cats.

## Supplementary information

Appendix 1

Appendix 2

## Data Availability

The datasets generated during and/or analysed during the current study are available from the corresponding author on reasonable request.
