# Supplementary material for: Facial expressions of pain in cats: the development and validation of a Feline Grimace Scale
Source: Sci Rep. 2019 Dec 13;9:19128. doi: 10.1038/s41598-019-55693-8 (PMC6911058; doi:10.1038/s41598-019-55693-8)
Supplement: Supplementary file 1 — Appendix 1 [file 41598_2019_55693_MOESM1_ESM.pdf]

Supplementary material

Facial expressions of pain in cats: the development and validation of a Feline Grimace Scale

Marina C Evangelista, Ryota Watanabe, Vivian SY Leung, Beatriz Monteiro, Elizabeth O’Toole, Daniel SJ Pang, Paulo V Steagall

TRAINING MANUAL

FELINE GRIMACE SCALE

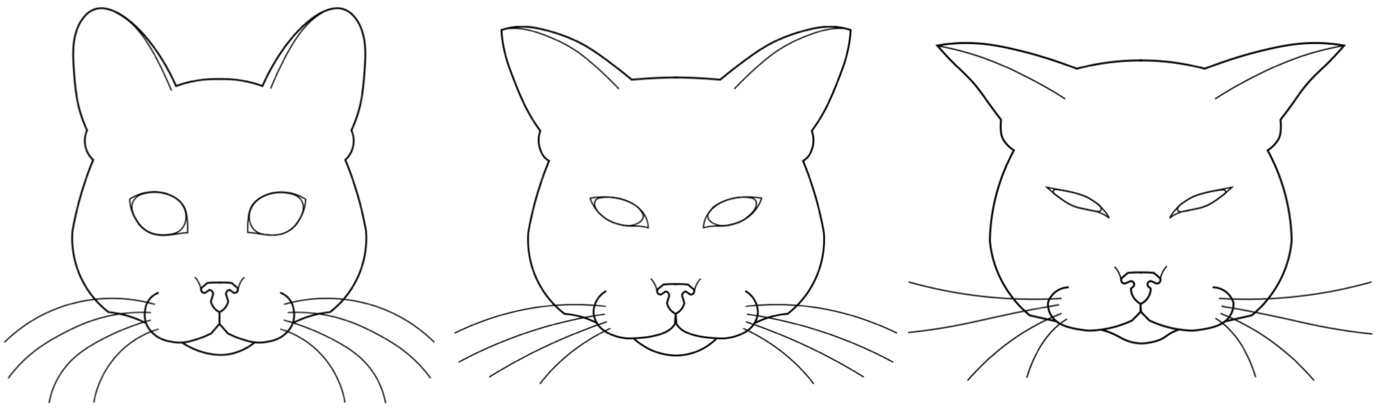

Instructions for using the scale

Rate each action unit from 0 to 2:

0 = action unit is absent
1 = moderate appearance of the action unit, or uncertainty over its presence or absence
2 = obvious appearance of the action unit

If the action unit is not visible, please mark the option “not possible to score”

Ear position

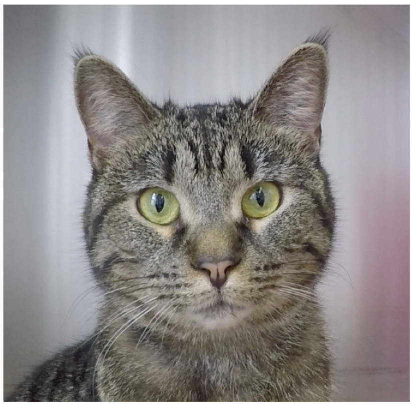

0 = absent

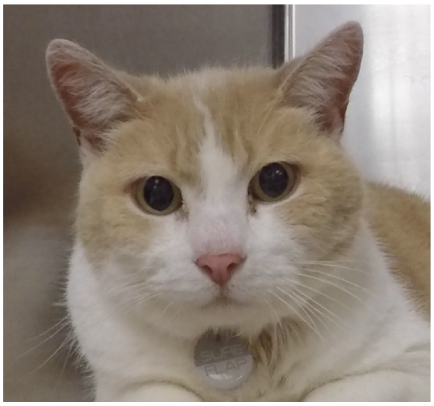

1 = moderately present

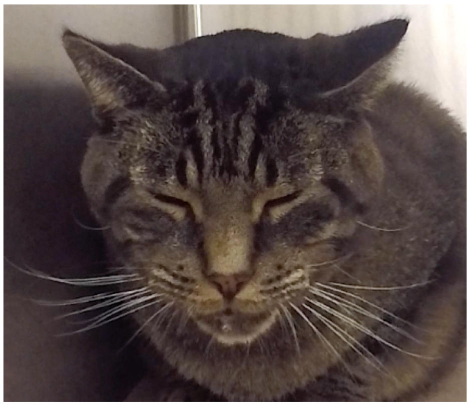

2 = markedly present

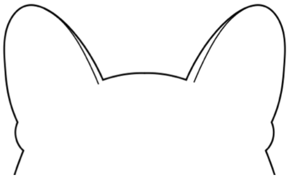

Ears facing forward

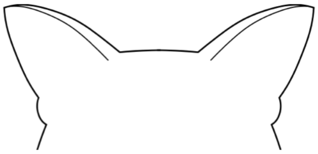

Ears slightly pulled apart

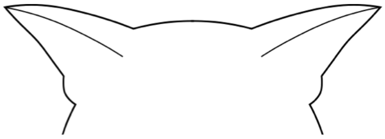

Ears rotated outwards

Orbital tightening

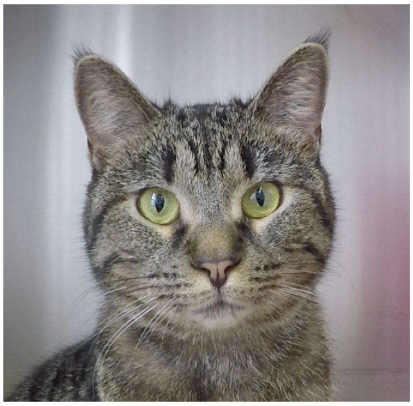

0 = absent

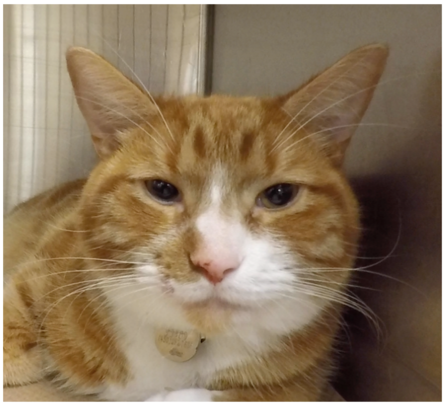

1 = moderately present

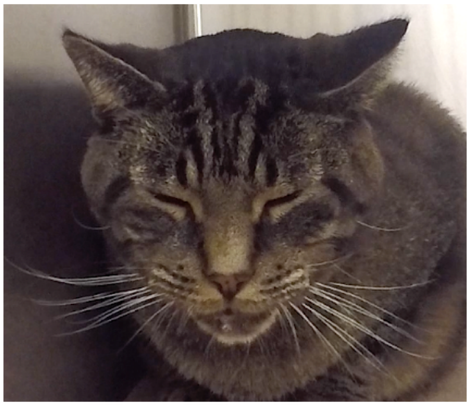

2 = markedly present

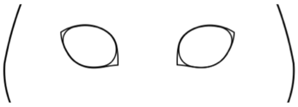

Eyes opened

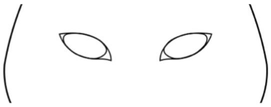

Partially closed eyes

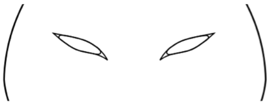

Squinted eyes

Muzzle tension

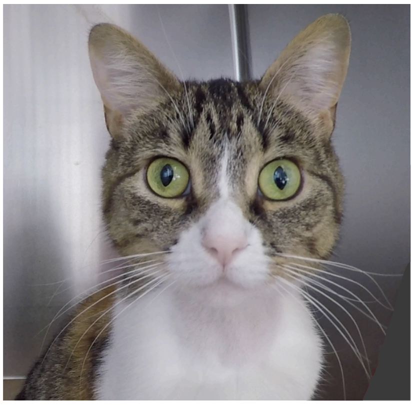
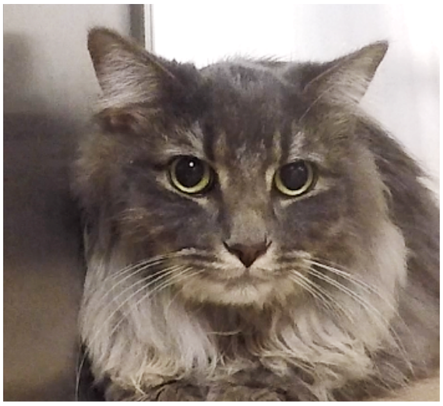
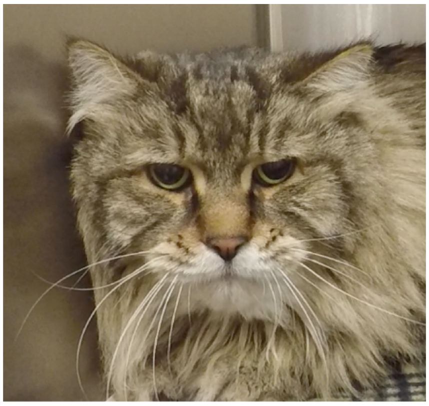

0 = absent | 1 = moderately present | 2 = markedly present

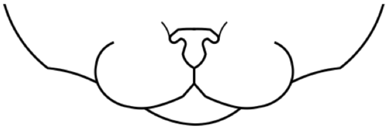
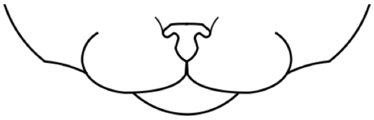
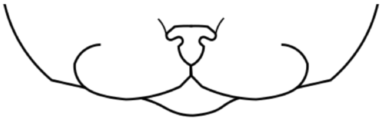

Relaxed (round shape) | Mild tension | Tense (elliptical shape)

Whiskers change

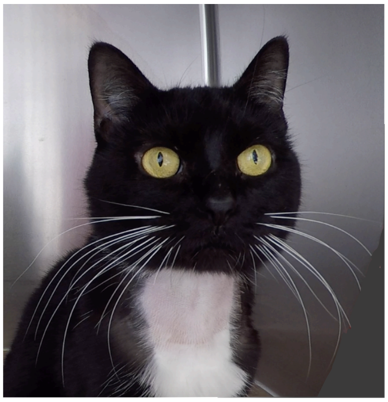
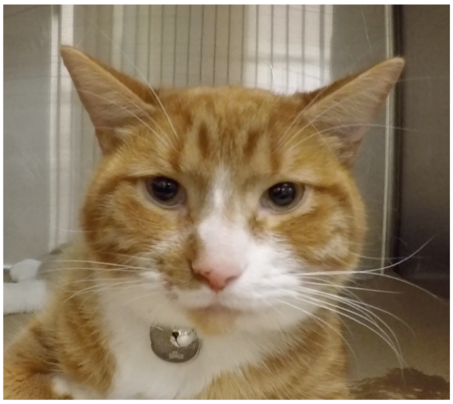
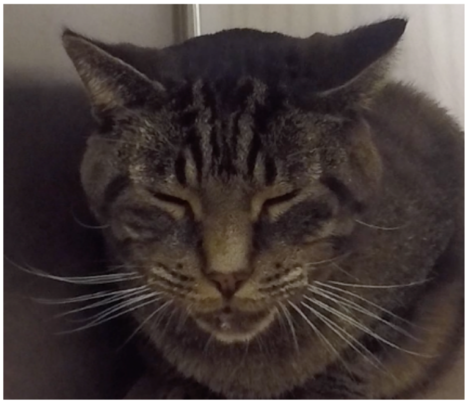

0 = absent | 1 = moderately present | 2 = markedly present

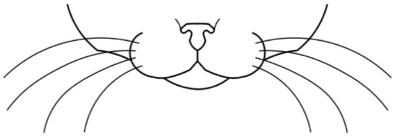
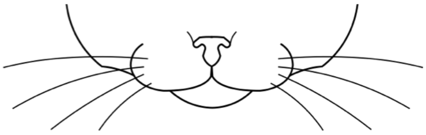
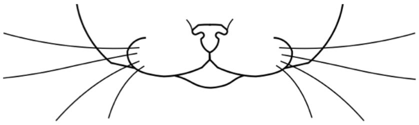

Loose (relaxed) and curved | Slightly curved or straight (closer together) | Straight and moving forward (rostrally, away from the face)

Head position

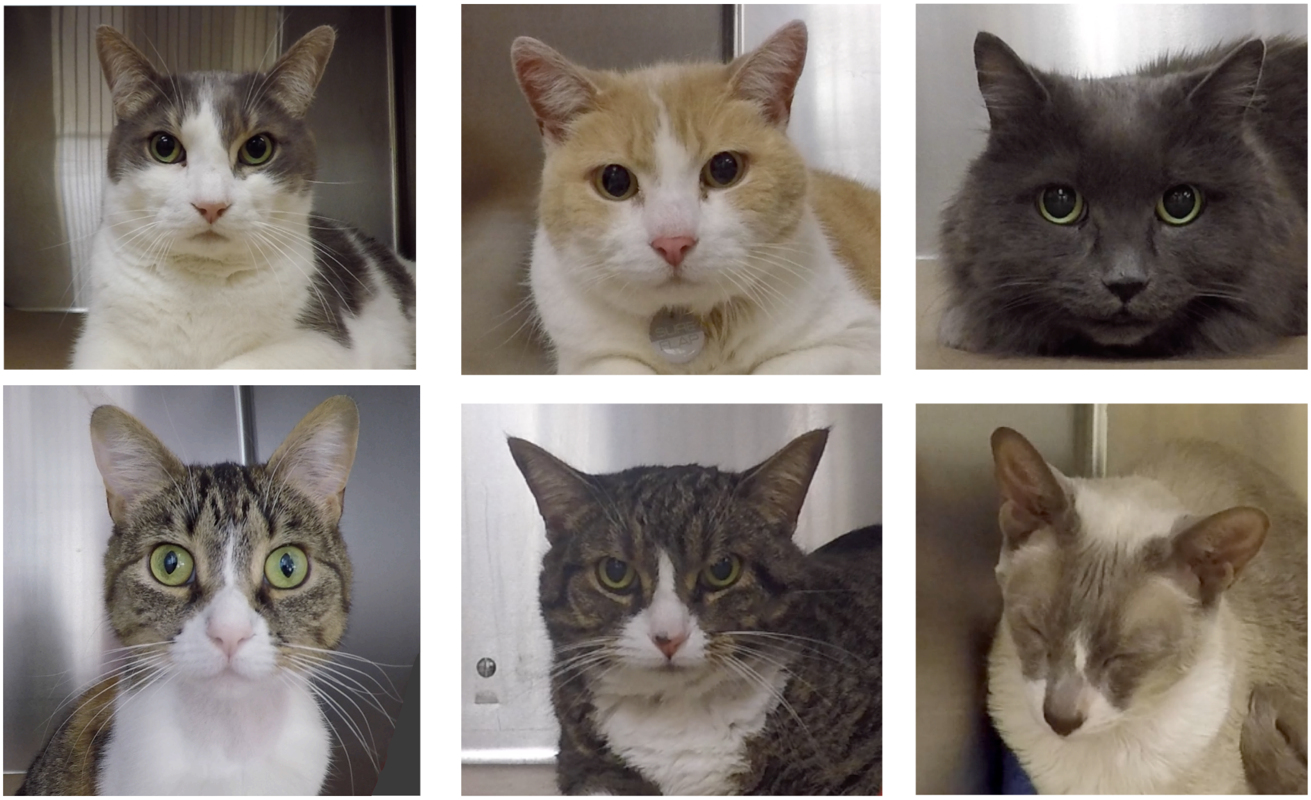

0 = absent

1 = moderately present

2 = markedly present

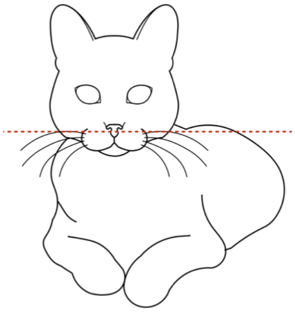

or

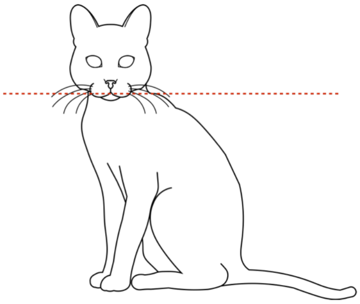

Head above the shoulder line

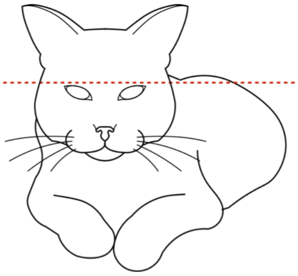

Head aligned with the shoulder line

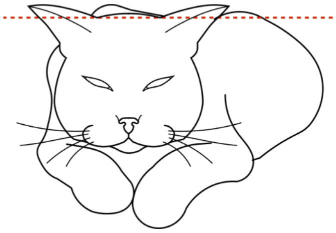

or

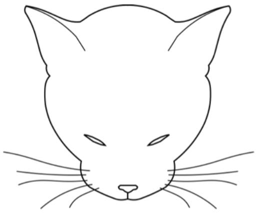

Head below the shoulder line or tilted down (chin toward the chest)
