# Supplementary material for: Facial expressions of pain in cats: the development and validation of a Feline Grimace Scale
Source: Sci Rep. 2019 Dec 13;9:19128. doi: 10.1038/s41598-019-55693-8 (PMC6911058; doi:10.1038/s41598-019-55693-8)
Supplement: Supplementary file 2 — Appendix 2 [file 41598_2019_55693_MOESM2_ESM.pdf]

Supplementary material – appendix 2

Facial expressions of pain in cats: the development and validation of a Feline Grimace Scale

Marina C Evangelista, Ryota Watanabe, Vivian Leung, Beatriz Monteiro, Elizabeth O'Toole, Daniel SJ Pang, Paulo V Steagall

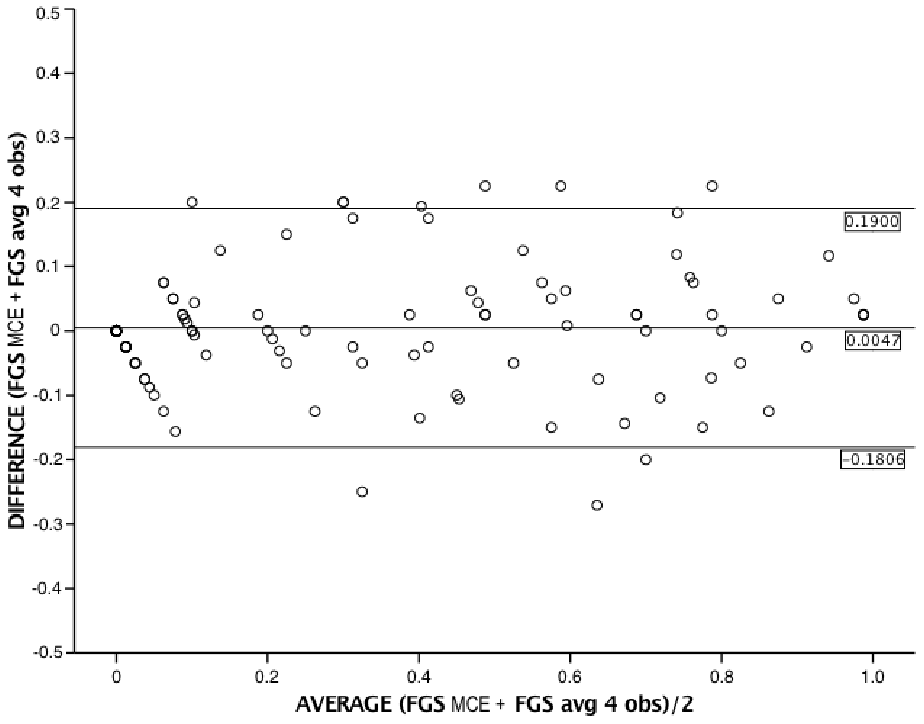

Figure S1. Bland and Altman plot comparing scores from the main observer (FGS MCE) and the four raters (FGS avg 4 obs) using the Feline Grimace Scale (FGS). The plot indicates minimal bias minimal bias (0.0047) and narrow limits of agreement, ranging from -0.18 to 0.19.

Table S1. Analgesic thresholds and their sensitivity and specificity

Cut-off score	Sensitivity (%)	Specificity (%)
0.05	100.0	50.7
0.11	100.0	68.7
0.16	100.0	70.1
0.23	93.0	79.1
0.28	90.7	79.1
0.32	90.7	83.6
0.35	90.7	85.1
**0.39***	**90.7**	**86.6**
0.45	81.4	91.0
0.55	65.1	94.0
0.61	48.8	95.5
0.65	46.5	95.5
0.68	44.2	95.5
0.73	34.9	97.0
0.78	32.6	97.0
0.82	18.6	98.5
0.87	16.3	98.5
0.95	11.6	100.0

*The cut-off for rescue analgesia of >0.39 was selected based on the highest values of sensitivity (90.7%) and specificity (86.6%). All the other cut-off values are the averages of two consecutive ordered observed test values.
